# Readability of Cancer Clinical Trials Websites

**DOI:** 10.1177/1073274819901125

**Published:** 2020-01-24

**Authors:** Grace Clarke Hillyer, Melissa Beauchemin, Philip Garcia, Moshe Kelsen, Frances L. Brogan, Gary K. Schwartz, Corey H. Basch

**Affiliations:** 1 Department of Epidemiology, Mailman School of Public Health, New York, NY, USA; 2 Herbert Irving Comprehensive Cancer Center, Columbia University, New York, NY, USA; 3 New York Presbyterian Hospital, New York, NY, USA; 4 Columbia University School of Nursing, New York, NY, USA; 5 Department of Public Health, William Paterson University, Wayne, NJ, USA

**Keywords:** readability, Internet, cancer, clinical trials, information seeking

## Abstract

Clinical trials are critically important for the development of new cancer treatments. According to recent estimates, however, clinical trial enrollment is only about 8%. Lack of patient understanding or awareness of clinical trials is one reason for the low rate of participation. The purpose of this observational study was to evaluate the readability of cancer clinical trial websites designed to educate the general public and patients about clinical trials. Nearly 90% of Americans use Google to search for health-related information. We conducted a Google Chrome Incognito search in 2018 using the keywords “cancer clinical trial” and “cancer clinical trials.” Content of the 100 cancer clinical trial websites was analyzed using an online readability panel consisting of Flesch-Kincaid Grade Level, Flesch Reading Ease, Gunning-Fog Index, Coleman-Liau Index, and Simple Measure of Gobbledygook scales. Reading level difficulty was assessed and compared between commercial versus non-commercial URL extensions. Content readability was found to be “difficult” (10.7 grade level). No significant difference in readability, overall, and between commercial and non-commercial URL extensions was found using 4/5 measures of readability; 90.9% of commercial versus 49.4% of non-commercial websites were written at a >10th grade (*P* = .013) using Gunning-Fog Index. Written cancer clinical trials content on the Internet is written at a reading level beyond the literacy capabilities of the average American reader. Improving readability to accommodate readers with basic literacy skills will provide an opportunity for greater comprehension that could potentially result in higher rates of clinical trial enrollment.

## Introduction

Clinical trials are critically important for the development of new cancer treatments; however, according to recent estimates, clinical trial enrollment is only 8%, ranging from 6.3% to 7.0% at community centers and between 14.0% and 15.9% at academic centers.^1-3^ Lack of awareness of and knowledge about clinical trials is one of the major barriers to patient participation.^4-6^ To close this gap in understanding, many cancer centers and clinical practices that offer clinical trials provide information such as what clinical trials are, their purpose, types of trials, the importance of considering trial participation, how safety is ensured, what to expect when enrolled in a trial, and how to join a trial to their patients through the most commonly used source of health-related information—the Internet.^7-9^

The National Institutes of Health (NIH) recommend that health-related materials be written at the seventh- to eighth-grade level^10^ to make information accessible to the approximately 43% of American adults who have basic or below basic literacy skills.^11^ Additional considerations for effective written communication, include the legibility of the material which relates to typography; the comprehensibility or how well the user can understand the intended meaning; and the readability of the material, that is, the ease at which the text can be read and understood.^12^ Comprehensibility and readability are perhaps the most important aspects of communicating complex written health-related material. Comprehensibility is more difficult to determine but readability can be assessed with a variety of formulas,^13^ each of which evaluate word and sentence length using different weighting factors.^14^ Readability formulas are objective and quantitative in nature and estimate the difficulty of written material by assessing a wide range of content and prose styles. However, because no formula is 100% accurate, the use of more than 1 readability formula when evaluating written content is preferable to improve the validity of the results.^15^

Evaluation of online sources of cancer-related information (eg, screening, treatment)^16-19^ has found that information is written at reading levels above the sixth- or seventh-grade level and thus beyond the ability of the average reader. An assessment of 165 988 trials registered as ClinicalTrials.gov^20^ through 2014 reported that, on average, 18 years of education (Master's level) are needed to properly understand the trial descriptions using 4 independent readability algorithms.^20^ A review of the top 100 cancer clinical trial websites on Google and Yahoo in 2005 found that the overwhelming amount and diversity of information as well as the complex language used was a deterrent to patients with cancer.^21^ Other examinations of the readability of Internet-based information about clinical trials has been confined to the understandability of recruitment resources,^22^ eligibility criteria,^23^ and informed consent.^24^ The purpose of this study was to evaluate the readability of cancer clinical trial websites and replicate the experience of the average American searching for information about cancer clinical trials using a systematic search of the top 100 clinical trial websites available on Google Chrome Incognito in 2018 and applying a panel of validated readability tests.

## Methods

Google Chrome is the most commonly used search engine in the United States and worldwide, accounting for 88.25% of the search engine market share in the United States and 92.78% around the world, followed by Bing (6.33% United States and 2.55% worldwide) and Yahoo (3.84% United States and 1.61% worldwide).^25^ Using the Google Chrome Incognito web browser to assure generality of the returned results, the search terms “cancer clinical trial” and “cancer clinical trials” were entered. Unlike Google Chrome, Google Chrome Incognito does not save browsing history, cookies, and site data and, therefore, is not influenced by prior search history and cookies on the device used to collect study data when displaying the search results. The first 100 websites that were active in 2018, in the English language, and that provided general information about cancer clinical trials were included. Industry research indicates that the first search engine results page (SERP) receives almost 95% of web traffic and the 67% of all clicks on the first page go to the top 5 listings.^26^ Therefore, to include all possible cancer clinical trial websites, we searched the first 20 SERPs which yielded 100 websites. Excluded were websites that collected contact information for the purpose of enrollment and search sites to locate a cancer clinical trial based on the entry of specific clinical criteria. The search was conducted by a single researcher (G.C.H.).

To calculate the scores with the Flesch-Kincaid Grade Level (FKGL), Flesch Reading Ease (FRE), Gunning-Fog Index (GFI), Coleman-Liau (CLI), and Simple Measure of Gobbledygook (SMOG) tests, an online calculator,^27^ recommended by the NIH^28^ and used in research by others when evaluating online health information,^29-33^ was employed. The online readability calculator evaluates the page found at the URL provided using the series of readability formulas selected. The presentation of the text (eg, headings, bullets) is taken into account and all material on the page is “read” which is consistent with the manner in which a reader would view the site. Any pages without text were excluded. In the case when a formatting error was encountered, text was manually entered into the calculator and scores were generated. To calculate scores, the FKGL and FRE tests both utilize the average sentence length and average syllables per word,^34,35^ whereas GFI assesses average sentence length and the use of polysyllabic words.^36^ CLI calculates the average number of letters per 100 words and the average sentence length,^37^ and SMOG evaluates number of polysyllabic words in 3 ten-sentence samples^38^ to determine the US academic grade level at which written material can be comprehended. The FRE is scored on a 100-point scale with scores of 0 to 29 considered “very confusing,” 30 to 49 “difficult,” 50 to 59 “fairly difficult,” 60 to 69 “standard” (eighth- and ninth-grade level), 70 to 79 “fairly easy,” 80 to 89 “easy,” and 90 to 100 “very easy” (fifth-grade level) readability.^39^

### Data Analysis

For each readability score, the minimum and maximum scores were reported and the overall mean and standard deviation (SD) were calculated. Websites were classified as non-commercial or commercial based on the URL extension with .org, .gov, and .edu coded as non-commercial and .com, .net, or other coded as commercial. Scores for the FKGL, GFI, CLI, and SMOG tests were recoded as “easy, <grade 6,”^12^ “average, grade 6-10,” and “difficult or higher than grade 10.”^29-33^ Flesch Reading Ease scores were grouped as 80 to 100 = “easy,” 60 to 79 = “average,” and 0 to 59 = “difficult.” Differences in the mean scores and grade level, overall, and between non-commercial and commercial websites were computed using Student *t* test and χ^2^ test of association, respectively. Values of *P* <.05 were considered statistically significant. All analyses were performed using IBM SPSS version 25.^40^ This study was exempted from human subjects review by the Columbia University Institutional Review Board.

## Results

The mean grade level scores for all 5 tests ranged from 10.7 (SD: 1.9) to 12.9 (SD: 1.7); mean FRE was 41.5, indicating a mean readability at the “difficult, grade >10” reading level (Table 1). Six of the 100 websites analyzed were scored as “easy, grade <6” using the GFI test. Of the 100 websites evaluated, 89 were categorized as noncommercial (.org, .gov, and .edu) and 11 as commercial (.com, .net, and other) based on the URL extension (Table 2). Overall, the mean readability scores of noncommercial website and commercial sites did not differ using each of the 5 measures. When evaluating grade level differences, only 6 (6.7%) noncommercial websites and none of the commercial websites were written at the “easy, grade <6 level.” All websites categorized as noncommercial (range 10.7-12.2 grade level with FKGL, GFI, CLI, and SMOG; 41.8 with FRE) as well as those categorized as commercial (range 11.4-13.2 grade level with FKGL, GFI, CLI, and SMOG; 39.1 with FRE) by URL were written at a “difficult (grade >10)” reading level. Only the GFI found a statistically significant difference in the reading level between noncommercial and commercial websites with 49.4% versus 90.9% scored as difficult (*P* = .013), respectively.

**Table 1.** Readability Characteristics of Cancer Clinical Trial Websites.

Test	Number of Websites (n = 100)			Readability Score	
	Easy (Grade < 6)	Average (Grade 6-10)	Difficult (Grade > 10)	Mean (SD)	Range
FKGL	0	55	45	10.7 (1.9)	6.7-16.3
GFI	6	40	54	10.9 (2.9)	1.3-18.9
CLI	0	17	83	12.9 (1.8)	9.9-17.5
SMOG	0	20	80	12.3 (1.7)	8.5-17.5
FRE^a^	0	3	97	41.5 (11.8)	3.5-62.8

Abbreviations: CLI, Coleman-Liau; FKGL, Flesch-Kincaid Grade Level; FRE, Flesch Reading Ease; GFI, Gunning-Fog Index; SD, standard deviation; SMOG, Simple Measure of Gobbledygook.

^a^ FRE scored on a scale of 0 to 100.

**Table 2.** Comparison of Websites by URL Extension (Noncommercial vs Commercial), n = 100.

Test	Mean (SD)		*P*	Noncommercial URL (n = 89)			Commercial URL (n = 11)			*P*
	Noncommercial URL (n = 89)	Commercial URL (n = 11)		Easy (Grade < 6)	Average (Grade 6-10)	Difficult (Grade > 10)	Easy (Grade < 6)	Average (Grade 6-10)	Difficult (Grade > 10)	
				n (%)	n (%)	n (%)	n (%)	n (%)	n (%)	
FKGL	10.7 (2.0)	11.4 (1.3)	.21	0 (0.0)	51 (57.3)	38 (42.7)	0 (0.0)	4 (36.4)	7 (63.6)	.19
GFI	10.7 (3.0)	12.3 (1.9)	.09	6 (6.7)	39 (43.8)	44 (49.4)	0 (0.0)	1 (9.1)	10 (90.9)	.013
CLI	12.8 (1.9)	13.3 (1.2)	.38	0 (0.0)	17 (19.1)	72 (80.9)	0 (0.0)	0 (0.0)	11 (100.0)	.11
SMOG	12.2 (1.7)	13.2 (1.0)	.07	0 (0.0)	20 (22.5)	69 (77.5)	0 (0.0)	0 (0.0)	11 (100.0)	.08
FRE^a^	41.8 (12.3)	39.1 (7.0)	.49	0 (0.0)	3 (3.4)	86 (96.6)	0 (0.0)	0 (0.0)	11 (100.0)	.54

Abbreviations: CLI, Coleman-Liau; FKGL, Flesch-Kincaid Grade Level; FRE, Flesch Reading Ease; GFI, Gunning-Fog Index; SD, standard deviation; SMOG, Simple Measure of Gobbledygook.

^a^ FRE scored on a scale of 0 to 100.

## Discussion

Our analysis of 100 English language cancer clinical trial websites using a panel of 5 well-known readability tests demonstrated that the majority of these websites are written at a mean grade level well beyond the reading capabilities of the average American reader. The mean readability level ranged from grade 10.7 to 12.3 using FKGL, GFI, CLI, and SMOG and the mean FRE score was 41.5; all scores are interpreted as “difficult.” That much of the written information available on the websites evaluated, overall, and when stratified by URL (noncommercial vs commercial), may not be understandable by a large proportion of the public suggests a systemic problem with the communication of complex clinical trial information to readers. Given that most patients and caregivers seeking health information on the Internet are frequently attempting to supplement information given by a care provider and use Internet-acquired information as both a prologue and an epilogue to the conversations held with their provider,^41^ difficult readability of these cancer clinical trial websites represents a missed opportunity to engage patients and their families and to empower patients to make informed cancer treatment decisions.

Websites have evolved over time from static sources of information to dynamic applications that provide a broad range of information. A study by Weinreich et al found that users frequently browse webpages rapidly—even those with substantial content, and, at most, the average web user reads 28% of the words during a visit to any website.^42^ When those with limited literacy skills seek information online, they are usually looking for specific information and typically spend about only 15 seconds or less on a page.^43^ Internet users with limited literacy and poor health literacy skills struggle to decode challenging words and have problems remembering their meanings. They often try to read every word, particularly when reading something very important, but also tend to skip words or sections that are too difficult to read.^43^ Capturing the web reader’s attention while conveying complex clinical trial information in an understandable format to readers with basic literacy skills, therefore, poses a unique challenge to web writers.

In 2010, the federal government enacted the Plain Writing Act requiring all federal agencies to use “plain language.” Plain language is a style of communication that is clear and concise to help readers find the information that they need and better understand the information they find to meet their informational needs.^44^ Despite this mandate, readers continue to struggle with government-created clinical trials text. For example, the comprehensibility of clinical trial eligibility posted on ClinicalTrials.gov used for recruitment purposes among a lay audience was evaluated by Kang et al.^23^ Due to the frequent use of medical and technical jargon, the authors found that a college-level reading ability was required to understand the clinical trial eligibility text. An examination of the use of plain language in a cancer clinical trial website/app by Schultz et al found that the medically complex titles and descriptions of clinical trials on online applications also presents an enormous barrier to the general public.^45^ After creating plain language versions of the cancer type and basic inclusion/exclusion criteria of 10 trial descriptions and testing among 217 volunteers, users showed better comprehension of the inclusion/exclusion criteria but showed continued to experience challenges in comprehending the study treatment plan. Although plain language descriptions can help users to understand the basics about clinical trials, much work is still needed to effectively communicate the treatment being studied to increase user comprehension.^45^

In addition to clinical trial websites, other trial-related materials created for patients are often difficult to comprehend. Friedman et al assessed the readability of clinical trial recruitment materials (38% print and 62% web-based materials) and found the overall reading level was grade 11.7. Importantly, web-based materials were significantly more likely to be written at a higher grade level than printed materials.^22^ Storino et al investigated the readability and accuracy of online pancreatic cancer patient resources and found that websites devoted to pancreatic cancer clinical trials had a median readability score of 15.2 (interquartile range, 12.8-17.0, *P* = .002).^46^ In addition to other well-known barriers to clinical trial enrollment, the difficulty in understanding clinical trial information likely contributes to a lack of understanding about clinical trials and unwillingness to participate.

As with any study, ours have certain limitations. All clinical trial sites analyzed were identified using a Google search, the primary search engine in the world.^47^ Those seeking information about clinical trials, however, may use other engines such as Bing or Yahoo that serve millions of search queries per day and this may have influenced the selection of sites for this study. An analysis of the transaction log of ClinicalTrials.gov by Graham et al^48^ demonstrated that 69% of users begin their search for information related to clinical trials using a search engine despite the availability of high-quality domain-specific resources. The top-referring site was Google with 41% of users accessing clinical trial information in this way.^48^ This strategy, therefore, results in sites/pages indexed by the search engine as “most relevant” getting the most exposure and greatest number of direct visits. All websites evaluated were written in English and affiliated with organizations in the United States; therefore, our findings may not be generalizable to clinical trial websites outside the United States. Finally, we only examined readability of cancer clinical trial websites. Other measures such as cohesion, legibility, and comprehensibility were not evaluated and may provide additional insight into the usefulness of online information about cancer clinical trials.

## Conclusion

We consider our findings to be an important contribution to the literature given that the Internet is the most common source for individuals to find health-related information and that current rates of cancer clinical trial participation are low. Further, our findings are consistent with that of others who examined Internet-based clinical trial recruitment resources (eg, ClinicalTrials.gov and others), patient resources, clinical trial eligibility criteria, and informed consent and, similarly, found that a high-grade level reading ability is required to comprehend clinical trial information.^22-24^

Simplifying the readability of cancer clinical trials information on the Internet to accommodate readers with basic literacy skills and augmenting written materials with videos, pictures, and FAQ sheets to actively engage patients in learning about cancer clinical trials^49^ would assist in increasing the accessibility of clinical trial information to nearly half of the US population and provide an opportunity for greater understanding that could potentially result in higher rates of enrollment into clinical trials.
